# Diabetic Ketoacidosis Complicated by a Brain Death

**DOI:** 10.7759/cureus.8903

**Published:** 2020-06-29

**Authors:** Sohaip Kabashneh, Zaynab Al-Sagri, Samer Alkassis, Layla Shanah, Hammad Ali

**Affiliations:** 1 Internal Medicine, Wayne State University/Detroit Medical Center, Detroit, USA

**Keywords:** type i diabetes mellitus, severe diabetic ketoacidosis, cerebral edema

## Abstract

Diabetic ketoacidosis (DKA) is a life-threatening complication of diabetes mellitus (DM). Cerebral edema (CE) can complicate DKA management. We report a patient with no significant medical history who presented with DKA and a new-onset DM; she received the standard management with regular insulin and IV fluids, the management resulted in a rapid drop in serum osmolality, the patient`s mental status deteriorated and became nonresponsive, brain imaging confirmed CE, a few days later the patient was declared brain dead by neurology. This case highlights the importance of gradual correction of hyperosmolar conditions including hyperglycemia and urges all healthcare providers to closely trend glucose levels in the management of DKA.

## Introduction

Diabetic ketoacidosis (DKA) is an acute, life-threatening complication of diabetes mellitus (DM) occurring more commonly in type one DM. DKA is characterized by elevated blood glucose, anion gap metabolic acidosis, and ketonemia [1-3]. Osmotic diuresis due to hyperglycemia leads to volume depletion and electrolyte imbalance which are the hallmark of the disease process [3]. The backbone of management in DKA consists of fluid resuscitation, electrolyte repletion, and hyperglycemia correction by insulin infusion.

Hypoglycemia and hypokalemia are the most common complications of the treatment of DKA. However, cerebral edema (CE), although infrequent, can lead to devastating outcomes, especially in the younger population. We present a case of a young patient who presented with DKA; her management was complicated by a rapid drop in plasma osmolality and subsequently developed CE and brain death.

## Case presentation

We present a 20-year-old female with no previous medical history who was brought to the hospital by her parents. Two days prior she became nauseated and had one episode of emesis, then she became sleepy and subsequently became less responsive. On initial assessment, she was obtunded, had a temperature of 36.9°C, a heart rate of 110 beats per minute, a blood pressure of 100/55 mmHg, oxygen saturation of 98% on room air. On neurological assessment both pupils were three millimeters, equal and reactive to light, her eyes were open spontaneously, she was moving all her limbs spontaneously, but was not following command, she was also making incomprehensible sounds, not responding to questions. The examination of her lungs, heart, and abdomen was unremarkable.

Complete blood count revealed white blood cells 18.5 cells/µL (normal 3.5-10.6 cells/µL), hemoglobin 13.0 g/dL (normal 13.3-17.1 g/dL), and platelet 280 x 10^3 /microliter. Biochemical labs showed abnormalities consistent with DKA, beta-hydroxybutyrate 128 mg/dL (normal 0.2-2.8) (Table 1). Her labs also revealed acute kidney injury (AKI) with creatinine (Cr) of 4.54 mg/dL (normal 0.70-1.30) (Table 1). A urine drug screen was negative. Infectious workup with chest X-ray, COVID-19 nasal swab, urine analysis, urine culture, and blood culture were done but were unremarkable. She was started on an insulin drip and IV fluids for DKA, in less than 24 hours a repeat lab is done (Table 1). 

**Table 1:** The patient`s lab results on admission and 24 hours later. BUN, blood urea nitrogen

Labs	Day one 11:00 am	Day two 8:00 am
Blood glucose	2228 mg/dL	560 mg/dL
pH	6.91	7.25
HCO3	3 mEq/L	12 mEq/L
Anion gap	36 mEq/L	12 mEq/L
Sodium	125 mEq/L (corrected 159)	145 mEq/L (corrected 152)
BUN	60 mg/dL	38 mg/dL
Serum osmolality	461.7 mOsm/kg	345 mOsm/kg

**Figure 1:**
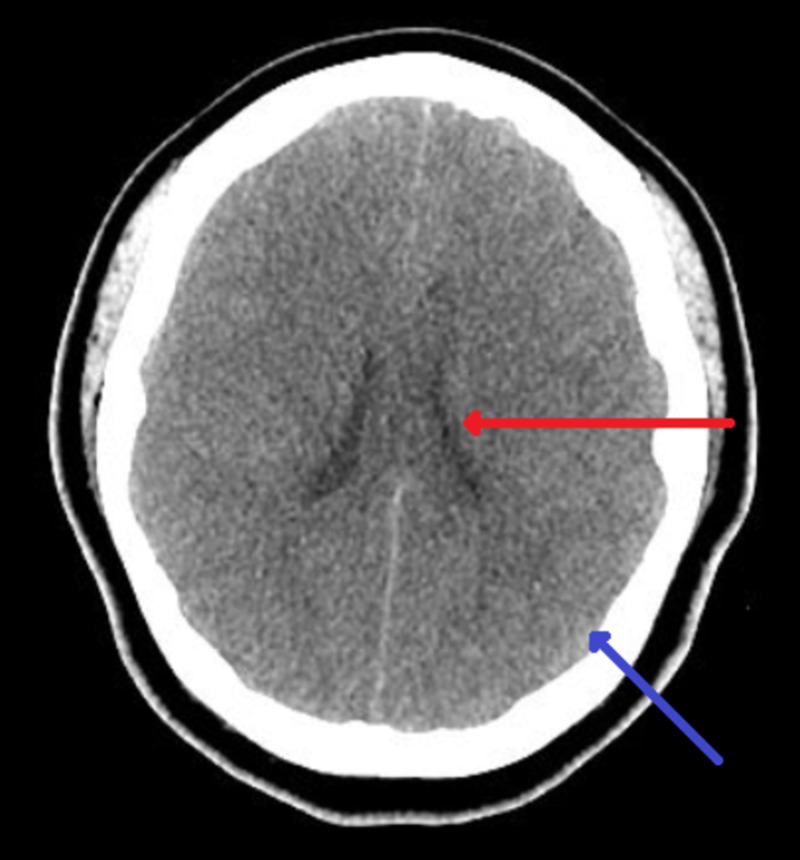
CT head showing the ventricles are slit like (red arrow) and the cortical sulci are diffusely absent (blue arrow), consistent with diffuse cerebral edema.

Later on the second day of admission, her mental status deteriorated further, she became none responsive, her Glasgow Coma Scale (GCS) dropped to three and she was subsequently intubated for airway protection and her ventilator settings were set at a respiratory rate of 18 breaths/minute, a tidal volume of 500 mL, a fraction of inspired oxygen of 100%, and a positive end-expiratory pressure of five cm H2O. CT scan of her brain was obtained quickly and showed changes consistent with CE (Figure 1). Neurology team was consulted, she had electroencephalography (EEG) done which showed moderate to severe degree of cerebral dysfunction consistent with metabolic/hypoxic encephalopathy, the waveforms were not epileptiform in nature. Despite multiple interventions including lowering PaCO2 to 25-30 mmHg, hypertonic saline 3%, pentobarbital coma, and cooling the patient to 35°C (95°F), she did not show any signs of improvement.

On the third day and fourth day of admission, her condition continued to worsen, and so on the fifth day and because she was not exhibiting response to painful stimuli, no brainstem reflexes, neurology proceeded with a brain death exam, which she had no arousal to voice or sternal rub, no withdrawal to painful stimuli, no cough response, gag or corneal reflexes, her pupils were dilated and fixed, nonreactive to light.

## Discussion

Symptomatic CE occurs in 1% of DKA cases [4]. However, subclinical edema occurs in almost half of DKA patients and can be detected by brain imaging [5]. The affected population is usually younger than 20 years with most cases occurring in DKA [6]. The most common risk factors for developing CE are newly diagnosed DM, young age, first episode of DKA, severity of DKA, and administration of bicarbonate [3].

Symptoms develop within 12-24 hours of the initiation of treatment but may be present before the onset of management. The first manifestation is a headache, followed by lethargy and decreased arousal. Seizures, incontinence, pupillary changes, bradycardia can develop with the worsening of edema. CE is a life-threatening complication, brain herniation is the leading cause of death in DKA-associated CE, it has a mortality rate of 20%-40% [7]. 

Cerebral edema develops when fluid moves from the extracellular to the intracellular compartment faster than the brain cells can adapt to increased intracellular volume [8]. This can happen when there is rapid correction of hyperglycemia, leading to a sudden drop in serum osmolality. The pathophysiology of CE in hyperglycemic crises is not fully understood. The correction of hyperglycemia is usually accompanied by a concurrent rise in serum sodium, which ameliorates the rapid drop in serum osmolality if blood glucose is corrected isolation. Another mechanism involves idiogenic osmoles, which are osmotically active substances produced within brain cells during periods of extracellular hyperosmolality to counteract the osmolar imbalance. These idiogenic osmoles have a slow clearance rate, resulting in high intracellular osmoles which can lead to the movement of extracellular fluid into the intracellular space [8].

In order to reduce the risk of CE in high-risk patients, in 2009 the American Diabetes Association (ADA) recommended the following measures to reduce the risk of CE in high-risk patients [9]:

1) Gradual replacement of sodium and water deficits in patients who are hyperosmolar. The usual fluid regimen with isotonic saline can be started at a rate of 15-20 mL/kg/h with a maximum of <50 mL/kg in the first two to three hours.

2) Avoidance of rapid reduction of plasma osmolarity, and a gradual decrease in serum glucose. Dextrose should be added to the saline solution once the serum glucose levels have fallen to 200 mg/dL in DKA, the serum glucose should be maintained at 250-300 mg/dL until the patient's serum osmolality has normalized.

There is limited data regarding the effectiveness of CE treatment in adults. Recommendations depend on clinical judgment without scientific evidence. Small series in children suggest benefit from prompt administration of mannitol (0.25-1 g/kg) [10]. Some case reports suggest the use of hypertonic (3%) saline (5-10 mL/kg over 30 minutes) as an alternative to mannitol [11-12]. Intubation may be indicated for airway protection, hyperventilation (PCO2 < 22 mmHg) in those patients has been associated with worse outcome, therefore, should be avoided unless necessary [13].

## Conclusions

Diabetic ketoacidosis is associated with an increase in serum osmolality. The management typically includes insulin drip with a goal to decrease blood glucose and close the anion gap. Rapid correction of blood glucose and serum osmolality can lead to CE, particularly in children and adolescents. Slow correction and close monitoring of serum glucose and osmolality is essential to avoid such a devastating event.
